# Nonlinear Frequency Offset Beam Design for FDA-MIMO Radar

**DOI:** 10.3390/s23031476

**Published:** 2023-01-28

**Authors:** Yanjie Xu, Chunyang Wang, Guimei Zheng, Ming Tan

**Affiliations:** College of Air and Missile Defense, Air Force Engineering University, Xi’an 710051, China

**Keywords:** nonlinear frequency offset, FDA-MIMO radar, Chebyshev window based frequency offset, array structure design

## Abstract

The beam pattern of frequency diversity array (FDA) radar has a range–angle two-dimensional degree of freedom, which makes it possible to distinguish different targets from the same angle and brings a new approach to anti-jamming of radars. However, the beam pattern of conventional linearly frequency-biased FDA radar is range–angle-coupled and time-varying. The method of adding nonlinear frequency bias among the array elements of the FDA array has been shown to eliminate this coupling property while still allowing for better beam performance of the emitted beam. In this paper, we obtain a decoupled and time-invariant beam direction map using the FDA-multi-input–multi-output (FDA-MIMO) radar scheme and then obtain a sharp pencil-shaped main sphere beam pattern with range–angle dependence using a linear frequency offset scheme weighted by a Chebyshev window. Finally, the anti-interference performance of the proposed method is verified in an anti-interference experiment.

## 1. Introduction

Since the first introduction of FDA radars in [1], the important property that their beam patterns are range-dimension dependent and can vary with time has been discovered, which has attracted extensive attention from scholars home and abroad. When the time and frequency offsets are fixed, the emission direction pattern of the frequency-controlled array has a range–angle-dependent property, which is more flexible compared with the conventional phased array and has a very high potential in practical applications [2,3,4,5,6]. However, the conventional fixed-frequency offsets FDA emission direction diagram is “S” shaped, with range–angle coupling characteristics, and is time-varying, which is unfavorable for the practical application of radar. With the exploration of the nature of the FDA directional pattern characteristics, many beam design methods for FDA radars have emerged. The range–angle coupling characteristics of FDA radar is caused by introducing the frequency offset among the array elements, so the frequency offset also needs to be designed and optimized to solve the coupling problem. The first problem to be solved is the time-varying problem. The literature [7] studied the time-varying problem of FDA radar and designed a time-invariant beam pattern, and the literature [8,9] combined FDA radar with MIMO technology to form a time-invariant beam pattern as well. On the basis of the existing research, the literature [10] explored the fundamental reason for the time-varying nature of the beam pattern from the nature of FDA radar and proposed the concept of “time figure”, which is of guidance for FDA radar research. After a period of research, the FDA-MIMO radar regime was proved to be one of the available methods to solve the beam patterns’ coupling problems [11,12]. After solving the time-varying problem, the beam performance is the next issue to be considered. On the basis of ensuring the time invariance, many beam design schemes have been proposed to provide a more superior performance. A logarithmic frequency offset scheme is proposed in the literature [13], which solves the periodic problem in space and forms a point beam, but with poor performance. The literature [14] uses a particle swarm optimization algorithm to determine the frequency offsets and array element spacing to form a point-like transmit beam map, which also has a lower side flap in the range dimension, but the calculation is too complex. As studies proceeded, the design of FDA radar frequency offsets using a window function weighting scheme became popular. However, window function weighting schemes based on fixed windows have been studied more often, while window function weighting schemes based on flexible windows are still less mentioned [14,15,16,17,18,19,20,21,22]. Compared with the fixed window function, the flexible window function can have more degrees of freedom in the power proportion of the main and side flaps, and, therefore, a better performance of the beam direction map can be obtained [23]. Overall, the present research on frequency-controlled array transmit beam formation can basically solve the problems of coupling and periodicity, but the present beam design scheme still has the problems of insufficient beam main flap focus, low beam resolution, and high side flap, which is more unfavorable for the specific application of radar. Therefore, it is necessary to design a point-type transmitting beam with narrow main flap and low side flap. For linear frequency bias, the beam direction map is not point-like, which is not favorable for radar detection, and because of this, a frequency bias design is needed to improve the beam direction map performance. In this paper, the time-invariant FDA beam direction diagram is obtained on the basis of the FDA-MIMO radar system, and the beam is further optimized by using the Chebyshev window to weight the linear frequency offsets on the basis of the existing nonlinear frequency offsets schemes, obtaining the results of narrowing the main flap and reducing the side flaps of the transmit beam. To better test its application capability, it is combined with the Minimum Variance Distortion-free Response (MVDR) adaptive beamforming algorithm to test its performance in jamming immunity. The simulation results show that this scheme has a more obvious advantage in the beam pattern performance compared with several window function weighted frequency offset schemes that have been proposed.

## 2. System Model

**Figure 1:**
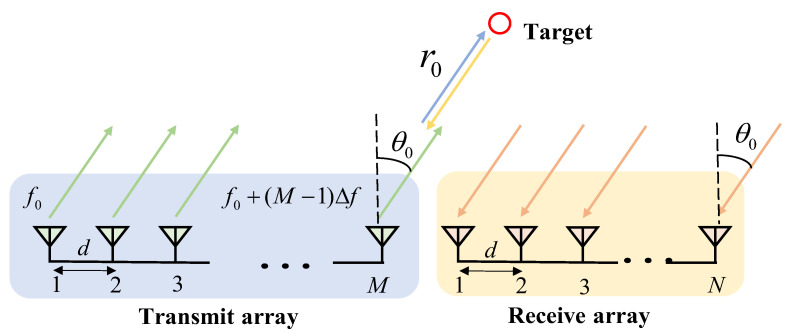
FDA-MIMO Radar System.

From the literature [10], we know that an effective measure to eliminate the time parameter in the transmit–receive diagram is to use a series of mixers and matched filters at the receiver. Analogously to this case, we can also use a combination of FDA-MIMO radar and multiple matched filters at the receiver side to produce beam maps independent of the time parameters. Consider a narrow-band FDA-MIMO radar system with M transmitting and N receiving arrays, and design both transmitting and receiving arrays as conventional linear arrays. Suppose the range between transmitting arrays is $d_{T}$, the range between receiving arrays is $d_{R}$, and the signal carrier frequency is $f_{0}$; such a system configuration is shown in Figure 1.

As shown in the figure, the frequency offset of the *m*th element is *f*(*m*), and the emission frequency of the *m*th array element can be expressed as

(1) $$f_{m}=f_{0}+f(m)$$

Suppose each array element emits the same waveform, then the signal emitted by the *m*th array element at time *t* can be expressed as Equation (2)

(2) $$s_{m}(t)=w_{m}(t)e^{j2\pi f_{m}t}$$

where $w_{m}(t)$ is the orthogonal signal envelope emitted by the *m*th array element, which satisfies

(3) $$\int w_{m1}{}^{*}(t)\cdot w_{m2}(t-\tau )dt=0,m1\neq m2.$$

where $w_{m1}{}^{*}(t)$ is the conjugate of the $w_{m1}(t)$. With the FDA-MIMO regime radar, the time delay at the target can be expressed as

(4) $$\tau_{m,n}=2r_{0}/c-md_{T}\sin\theta_{0}/c-nd_{R}\sin\theta_{0}/c$$

Then, the received signal related to the *m*th transmitting array element and the *n*th receiving array element can be expressed as

(5) $$s_{m,n}\left(t-\tau_{m,n}\right)=w_{n}\left(t-\tau_{m,n}\right)e^{j\Phi_{m}\left(t-\tau_{m,n}\right)}\cdot e^{j2\pi \left(f_{0}+f(m)\right)\left(t-\tau_{m,n}\right)}$$

where n=1,2,…,N, $\Phi_{m}$ is the phase modulation corresponding to the *m*th transmitting array element in the FDA-MIMO radar, and the time index can be expressed as

(6) $$t^{\prime }=t-\frac{2r}{c}$$

Then, under the far-field condition, Equation (2) can be rewritten as

(7) $$s_{m,n}\left(t^{\prime }\right)=w_{m}\left(t^{\prime }\right)e^{j\Phi_{m}\left(t^{\prime }\right)}e^{j\varphi_{n}\left(t^{\prime }\right)}$$

**Figure 2:**
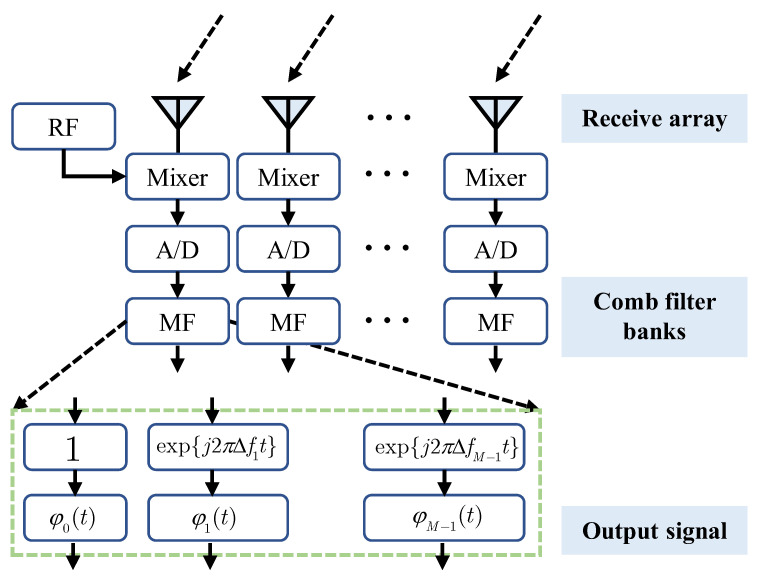
FDA-MIMO and multi-matching filter system.

In order to eliminate the effect of the time parameter and produce a time-invariant and decoupled transmit beam pattern, we need to use a multi-matching filter approach to process the signal at the receiver side of the FDA-MIMO radar, as shown in Figure 2.

As shown in the figure, the received signal needs to be mixed with the radio frequency of the mixer and later mixed with $w_{n}(t^{\prime })$ in the digital signal processor, so that the relative outputs of the *m*th transmitting array element and the *n*th receiving array element can be expressed as

(8) $$s_{m,n}^{\mathrm{Output}}\left(t^{\prime }\right)=\xi_{s}e^{j\Phi_{m}\left(t^{\prime }\right)}e^{j\varphi_{n}(t^{\prime })}$$

where $\varphi_{n}(t^{\prime })$ can be expressed as

(9) $$\begin{array}{l} \varphi_{n}\left(t^{\prime }\right)=2\pi \int_{0}^{t^{\prime }+\left(\left(md_{T}\sin\theta /c\right)+\left(nd_{R}\sin\theta /c\right)\right)}\left(f_{0}+f(m)\right)dx\\ =2\pi \left((f_{0}+f(m))t^{\prime }+f_{0}\frac{md_{T}\sin\theta }{c}+f_{0}\frac{nd_{R}\sin\theta }{c}\right) \end{array}$$

If the radar is aimed at the target at $\left(r_{t},\theta_{t}\right)$, the array factor AF can be expressed as

(10) $$\begin{array}{l} AF=\overset{M}{\underset{m=1}{\sum }}\overset{N}{\underset{n=1}{\sum }}s_{m,n}^{\mathrm{output}}\left(t^{\prime }\right)=\xi_{s}e^{-j2\pi f_{0}((2r/c)+md_{T}\left(\sin\theta -\sin\theta_{t}\right)/c)}\\ \cdot \overset{M}{\underset{m=1}{\sum }}e^{j(4\pi /c)f(m)\left(r_{t}-r\right)}\overset{N}{\underset{n=1}{\sum }}e^{j\left(2\pi f_{0}nd_{R}\left(\sin\theta -\sin\theta_{t}\right)/c\right)} \end{array}$$

The transmit–receive normalized beam pattern can be expressed as

(11) $$B=B_{T}\cdot B_{R}={\left|\overset{M}{\underset{m=1}{\sum }}e^{j(4\pi /c)f(m)\left(r_{t}-r\right)}e^{j\left(2\pi f_{0}md_{T}\left(\sin\theta -\sin\theta_{t}\right)/c\right)}\right|}^{2}\cdot {\left|\overset{N}{\underset{n=1}{\sum }}e^{j\left(2\pi f_{0}nd_{R}\left(\sin\theta -\sin\theta_{t}\right)/c\right)}\right|}^{2}$$

It is worth noting that the transmit–receive beam pattern in the above formula can be equivalent to the multiplication of the transmit beam pattern and the receive beam pattern at the receiver, which can be represented by $B_{T}$ and $B_{R}$, respectively.

Equation (11) shows that the FDA-MIMO radar can rely on its own characteristics to produce a time-invariant range-dependent beam pattern after the signal processing process, and that the beam pattern can be maximized at the target point with a maximum value of $M^{2}N^{2}$, regardless of the frequency offset change. Therefore, when designing the beam direction map of the FDA-MIMO radar, the range dependence of the FDA-MIMO radar should be activated by adding the corresponding variables of phase and target spatial position at the transmitter side.

## 3. Chebyshev Window Weighted Linear Frequency Offsets

For the window function weighted frequency offsets scheme, the parameter selection of the window function is an important element affecting the beam performance. Most of the window functions of variable windows are controlled by two parameters, so it is necessary to use the control variable method to find the best combination of parameters. Ordinarily, when choosing the best position for the performance of a parameter, it is not possible to make both the main flap width and the side flap height optimal. For example, when we set the parameter of the peak side flap of the Chebyshev window to a low level, we can ensure that the side flap energy is low, but the energy will be concentrated in the main flap, resulting in a wide main flap, which we do not expect. When we are designing a beam, we usually give priority to minimizing the width of the main flap if the flap level does not seriously affect the detection. On this basis, the Chebyshev window is utilized for frequency offset design in this paper. The advantage of the Chebyshev window function is that the main flap width of the Chebyshev window is minimum for a given flap height, and all flaps have the same amplitude, so that the beam with minimum main flap width can be obtained by controlling the flaps within an acceptable range when choosing parameters. For nonlinear frequency offset, the proposed frequency offset methods are: log frequency offset, Hamming window based frequency offset (Ham-FDA), and sinusoidal-weighted frequency offset. Our proposed method is a weighted linear frequency offset based on the Chebyshev window, which outperforms the conventional method in both the main flap width and the side flap height. The Log-FDA frequency offset can be expressed as

(12) $$\Delta f_{m}^{\log}=\delta \cdot \log(m+1)$$

where δ is a constant, and $\Delta f_{m}^{}$ represents the frequency offset of the first transmitting array element. Similarly, the frequency offset of Ham-FDA can be expressed as

(13) $$\Delta f_{m}^{\mathrm{Ham}}=B\left\{0.54-0.46\cos\left[\frac{2\pi (m-(M+1)/2)}{M}\right]\right\}$$

where B is the bandwidth and requires that the number of array elements *M* should be odd.

The frequency shift of the Hamming window weighted linear frequency offsets (HL-FDA) scheme proposed in the literature [17] can be expressed as

(14) $$\Delta f_{m}^{HL}=m\Delta f\left\{0.54-0.46\cos\left[\frac{2\pi (m-(M+1)/2)}{M}\right]\right\}$$

The frequency offset of the proposed algorithm in this paper can be expressed as

(15) $$\Delta f_{m}^{proposed}=m\Delta fw_{c}(m)$$

where $w_{c}^{}(m)$ is the time domain expression for the Chebyshev window function, which can be expressed as [24]

(16) $$w_{c}(m)=\frac{\sum_{m=-N}^{S}W_{c}^{0}(n)\cos\left(\frac{2\pi }{N}mn\right)}{\sum_{m=-N}^{S}W_{c}^{0}(n)}$$

where $W_{c}^{0}(n)$ is the discrete spectral expression of the Chebyshev window, and S should be defined as S=(M−1)/2. Similarly, there are s=(m−1)/2. As with the Hamming window weighting scheme, it is also required here that the number of array elements *M* should be odd. The algorithm proposed in this paper can be seen in the form of a Chebyshev window combined with a linear array of arrays, where the output signal associated with the *m*th transmitting array element and the *n*th receiving array element after processing at the receiver side of the FDA-MIMO system, as shown in Equation (17). The array factor AF can be expressed as Equation (18).

(17) $$\begin{array}{l} s_{m,n}^{Output}(t^{\prime })\approx rect(\frac{t^{\prime }}{T_{p}})\xi_{s}\exp\{j2\pi [-f_{0}\frac{2r}{c}-m\Delta fw_{c}(m))\frac{2r}{c}\\ +f_{0}\frac{md_{T}\sin\theta }{c}+f_{0}\frac{nd_{R}\sin\theta }{c}]\}\\ =rect(\frac{t^{\prime }}{T_{p}})\xi_{s}\exp\{j2\pi [-f_{0}\frac{2r}{c}-m\Delta f\frac{\sum_{m=-N}^{N}W_{c}^{0}(n)\cos\left(\frac{2\pi }{N}mn\right)}{\sum_{m=-N}^{N}W_{c}^{0}(n)})\frac{2r}{c}\\ +f_{0}\frac{nd_{T}\sin\theta }{c}+f_{0}\frac{md_{R}\sin\theta }{c}]\} \end{array}$$

(18) $$\begin{array}{l} AF=\sum_{m=0}^{M-1}\sum_{n=0}^{2N_{1}}s_{m,n}^{Output}(t^{\prime })\\ \;\;=rect(\frac{t^{\prime }}{T_{p}})\xi_{s}\sum_{m=0}^{M-1}\sum_{n=0}^{2N_{1}}e^{j2\pi [-f_{0}\frac{2r}{c}-m\Delta fw_{c}(m))\frac{2r}{c}+f_{0}\frac{md_{T}\sin\theta }{c}+f_{0}\frac{nd_{R}\sin\theta }{c}]} \end{array}$$

The vector of orientation A can be expressed as Equation (19), where a and b are the transmitting and receiving guide vectors, respectively, which can be expressed as Equations (20) and (21).

(19) $$A=\xi_{s}\;a(r,\theta )⊗b(\theta )$$

(20) $$a(r,\theta )=\left[\begin{array}{c} 1\\ e^{j2\pi [f_{0}\frac{d_{T}\sin\theta }{c}-m(\Delta fw_{c}(m))\frac{2r}{c}]}\\ \vdots \\ e^{j2\pi [f_{0}\frac{2Md_{T}sin\theta }{c}-2M(\Delta fw_{c}(m))\frac{2r}{c}]} \end{array}\right]$$

(21) $$b(\theta )=\left[\begin{array}{c} 1\\ e^{j2\pi f_{0}d_{R}\sin\theta /c}\\ \vdots \\ e^{j2\pi f_{0}d_{R}(N-1)\sin\theta /c} \end{array}\right]$$

Then, the emission direction diagram $B_{T,R}$ can be expressed as

(22) $$\begin{array}{l} B_{T,R}={\left|w_{D}{}^{H}\cdot A\right|}^{2}\\ \;\;={\left|\sum_{n=0}^{2M}\exp\{j2\pi (f_{0}d_{T}m\frac{\sin\theta -\sin\theta_{0}}{c}-2m\Delta fw_{c}(m))\}\right|}^{2}\\ \;\;\times {\left|\sum_{n=0}^{N-1}\exp\{j2\pi f_{0}d_{R}n\frac{\sin\theta -\sin\theta_{0}}{c}\}\right|}^{2} \end{array}$$

## 4. MVDR Algorithm Model

In order to better test the practical application capability of the proposed frequency offsets scheme, it is combined with the adaptive beamforming algorithm to verify its beam output performance under the conventional MVDR algorithm. Adaptive beamforming is an important method to enhance the signal output signal-to-noise ratio in the array radar signal processing process. By attaching a weighting factor between each array element, it causes the array output power to be maximum in the desired direction and minimum in the jamming direction, by which the output signal-to-noise ratio can converge quickly at the jamming location, thus forming a zero null.

In the FDA-MIMO system, the desired signal, the dummy target, and the noise are statistically independent of each other, and the location of the desired target in the setup space is $\left(r_{0},\theta_{0}\right)$, The interfering signal is $i_{j}$, j is the number of jammings, jamming position can be set to $\left(r_{j},\theta_{j}\right)$, and noise n(t) is Gaussian white noise. The signal at the *k*th snap count can be expressed as Equation (23), and the output signal in the FDA-MIMO system can be expressed as Equation (24).

(23) $$x(t)=a\left(r_{0},\theta_{0}\right)s(t)+\overset{J}{\underset{j=1}{\sum }}a\left(r_{j},\theta_{j}\right)i_{j}(t)+n(t)$$

(24) $$\begin{array}{l} y(t)=w^{H}\left[a\left(r_{0},\theta_{0}\right)⊗b\left(\theta_{0}\right)\right]s(t)+w^{H}\overset{J}{\underset{j=1}{\sum }}\left[a_{0}\left(r_{j},\theta_{j}\right)⊗b_{n}\left(\theta_{j}\right)\right]i_{j}(t)+n(t) \end{array}$$

To keep the energy of the noise minimum while satisfying the constraints, the optimization function of MVDR can be expressed as

(25) $$\begin{array}{l} \min_{\omega }w^{H}Rw\\ \;s.t.\;\{\begin{array}{l} w^{H}a\left(r_{0},\theta_{0}\right)=1\\ w^{H}a\left(r_{j},\theta_{j}\right)=0 \end{array} \end{array}$$

where R is the jamming-plus-noise covariance matrix. The equation can be solved by the Lagrange multiplier method, the optimal weights of the array are obtained as Equation (26), and the output signal-to-jamming-noise ratio (SINR) can be expressed as Equation (27)

(26) $$w_{MVDR}=\frac{R_{}^{-1}a(r_{0},\theta_{0})}{a{\left(r_{0},\theta_{0}\right)}^{H}R_{}^{-1}a(r_{0},\theta_{0})}$$

(27) $$R_{SINR}=\frac{{\left|w^{H}a(r_{0},\theta_{0})\right|}^{2}}{w^{H}R^{-1}w-{\left|w^{H}a(r_{0},\theta_{0})\right|}^{2}}$$

Finally, incorporating the guidance vector into the above equation, we obtain

(28) $$R_{SINR}=\frac{{\left|w^{H}(1,e^{j2\pi [f_{0}\frac{d_{T}\sin\theta }{c}-m(\Delta fw_{c}(m))\frac{2r}{c}]},\ldots ,e^{j2\pi [f_{0}\frac{2Md_{T}\sin\theta }{c}-2M(\Delta fw_{c}(m))\frac{2r}{c}]})\right|}^{2}}{w^{H}R^{-1}w-{\left|w^{H}(1,e^{j2\pi [f_{0}\frac{d_{T}\sin\theta }{c}-m(\Delta fw_{c}(m))\frac{2r}{c}]},\ldots ,e^{j2\pi [f_{0}\frac{2Md_{T}\sin\theta }{c}-2M(\Delta fw_{c}(m))\frac{2r}{c}]})\right|}^{2}}$$

## 5. Simulation and Analysis

### 5.1. Simulation of Frequency Offset Scheme

**Figure 3:**
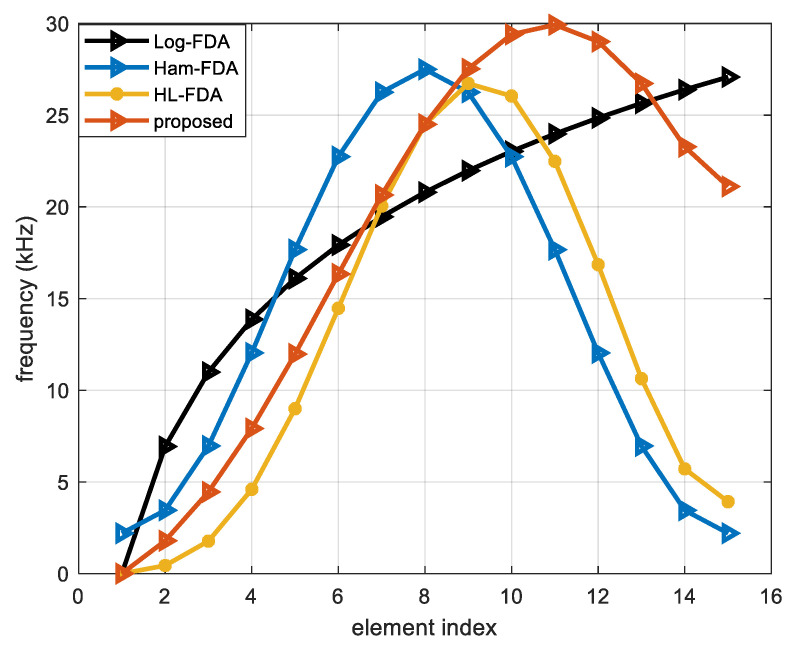
Relationship between array element index and position.

In our given FDA-MIMO system, the frequency offset affects only the equivalent transmit beammap and does not affect the receive beammap. Therefore, the simulation compares only the equivalent transmit beamplot. In this section, we simulate and compare the performance of Log-FDA, Ham-FDA, HL-FDA, and our proposed FDA frequency offset scheme. The maximum frequency offsets of these methods are set to be approximately equal, and the other relevant parameters are set as follows. The carrier frequency $f_{0}$ is 10 GHz, the array element index is M=N=15, the array spacing of the transmitting and receiving arrays is set to dt=dr=0.015 m, bandwidth is set to B=27.5 kHz, and the target position is set to $\left(100\;\mathrm{km},\;20^{{}^{\circ}}\right)$. Figure 3 compares the array element order versus position for each method.

**Figure 4:**
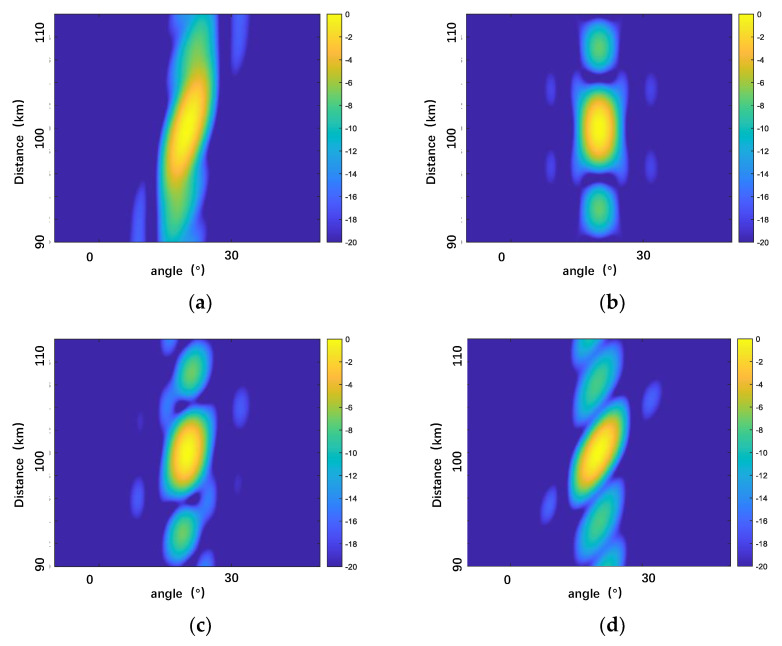
Equivalent transmitting antenna beams of the four schemes: (**a**) Log-FDA; (**b**) Ham-FDA; (**c**) HL-FDA; and (**d**) Proposed.

After designing the frequency offset on the FDA-MIMO system, we analyzed the equivalent transmit beamplots of four frequency offset schemes: Log-FDA, Ham-FDA, HL-FDA, and our proposed scheme, as shown in Figure 4.

**Figure 5:**
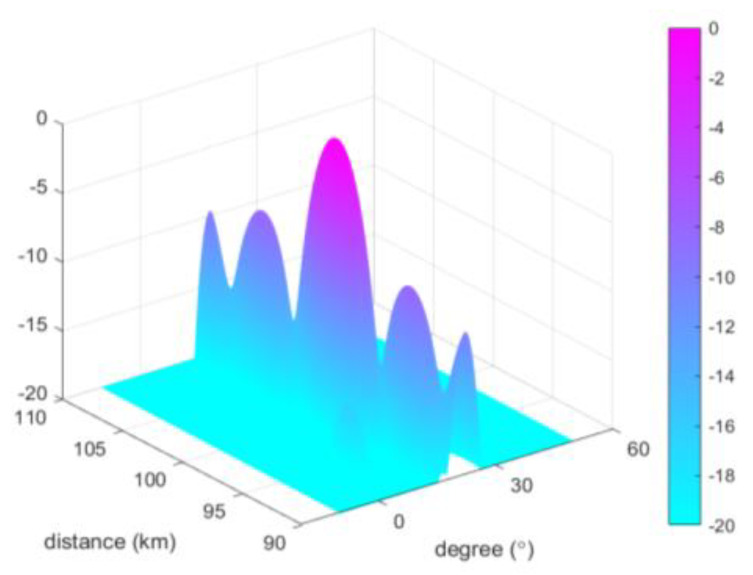
The 3D beam pattern.

As shown in Figure 4, several frequency offsets schemes have been proposed to solve the time-varying and coupling problems of the FDA directional map, but the beam performance varies. The Log-FDA scheme can solve the range–angle coupling problem, but the main flap of its formed beam is too wide and does not focus well, and the Ham-FDA and HL-FDA schemes can focus the emitted energy at a point in space. The Ham-FDA and HL-FDA schemes can produce a point-like beam, but the main flap of these two schemes is still very wide, and their side flaps are high in height, which also affects the radar detection performance to some extent. Our proposed method can produce a more focused point-like beam with a lower side flap than the other methods. The 3D view of the beam provides a more intuitive view of the overall effect of the beam, which is shown in Figure 5.

**Figure 6:**
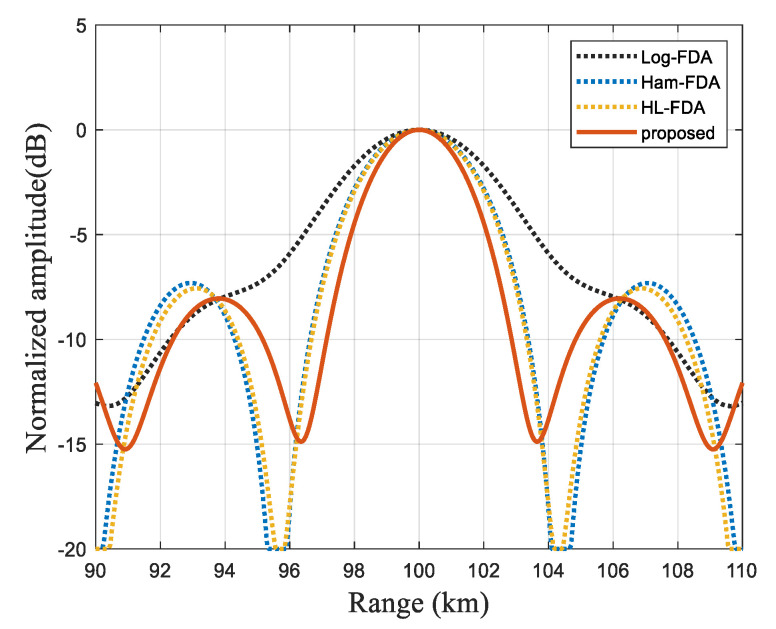
Range-Dimensional Slicing Performance Comparison.

As shown in Figure 5, it can be seen that our designed beam produces a lower side flap, which can help suppress the clutter in the side flap direction. It should be noted that, although our method produces a lower side flap, the depression energy between the main flap and the side flap becomes higher, which is the inevitable result of weighting the frequency offsets with a window function. However, this does not affect the detection because the side flap energy is still low compared with the main flap, and the jamming signal inside the side flap can be easily found during the detection process by aligning the main flap to the detection target. The range-dimensional slicing of the beam map can better reflect the performance of the beam, and the comparison of each method is shown in Figure 6.

**Figure 7:**
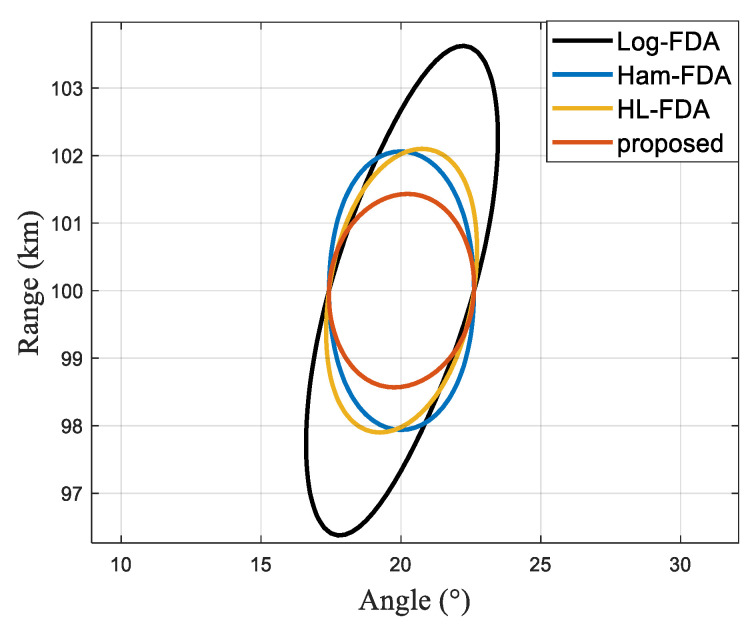
Half-power beamwidth diagram.

As shown in Figure 6, the proposed scheme has greater advantages at both the main flap and the side flap because the Chebyshev window function is used to evenly disperse the energy in the side flap to the airspace far away from the main flap. Compared with several proposed methods, our proposed scheme has better beam-focusing performance with a narrower main flap and good side flap height, and the half-power beamwidth diagram can be a good comparison of the performance gap of the scheme, as shown in Figure 7.

As represented in Figure 7, the half-power beamwidth comparison results show the superior performance of our proposed scheme in both angular and range dimensions.

### 5.2. Analysis of the Anti-Jamming Characteristics of the Proposed Scheme

**Figure 8:**
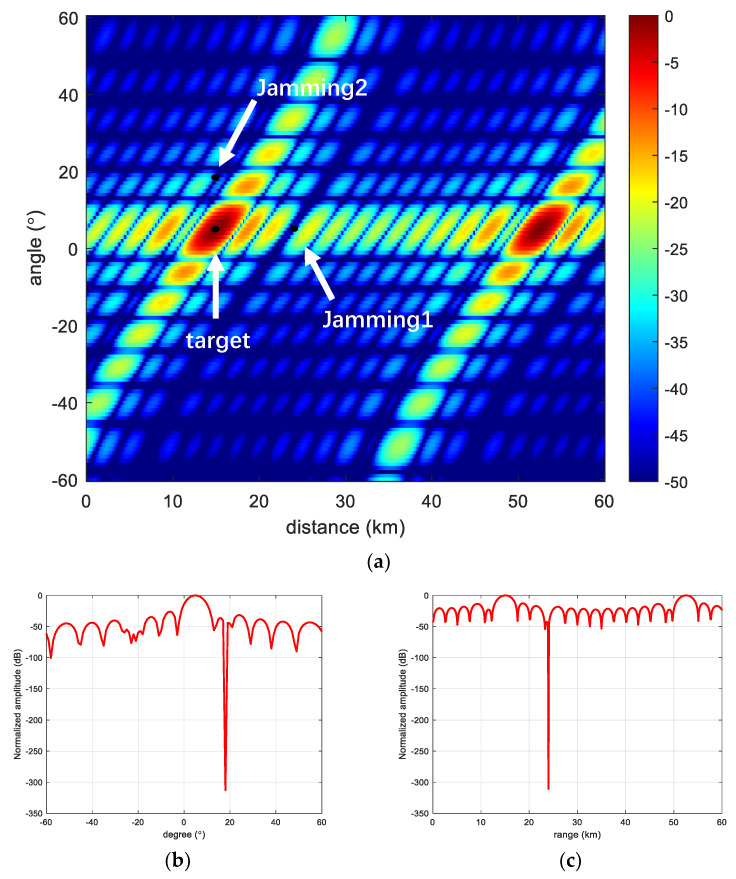
Linear frequency offset FDA-MIMO. (**a**) FDA-MIMO; (**b**) Range-dimensional slicing; and (**c**) Angle-dimensional slicing.

In this section, we verify the anti-jamming capability of the proposed frequency offsets scheme in the presence of jamming in space and verify its beam performance under MVDR conditions, respectively. In the experiment, a range-dimensional jamming and an angle-dimensional jamming are set up, respectively. It can be seen that Jamming 1 is at the same angle as the target but at a different range; it is located on the side flap of the range dimension, and its beam gain is lower than the gain at the target point. Jamming 2 is at the same range from the target but at a different angle; it is located in the angular dimension of the side flap, and its gain is also lower than that at the target point. Meanwhile, we can see from the range slice and angle slice that after the adaptive beam formation, the beam of FDA-MIMO radar will automatically form a null trap at the jamming to suppress the jamming, but for the linear frequency offsets FDA-MIMO, it still has more energy distribution in the angle dimension, which indicates its weak anti-jamming capability in the angle dimension. The details are shown in Figure 8.

**Figure 9:**
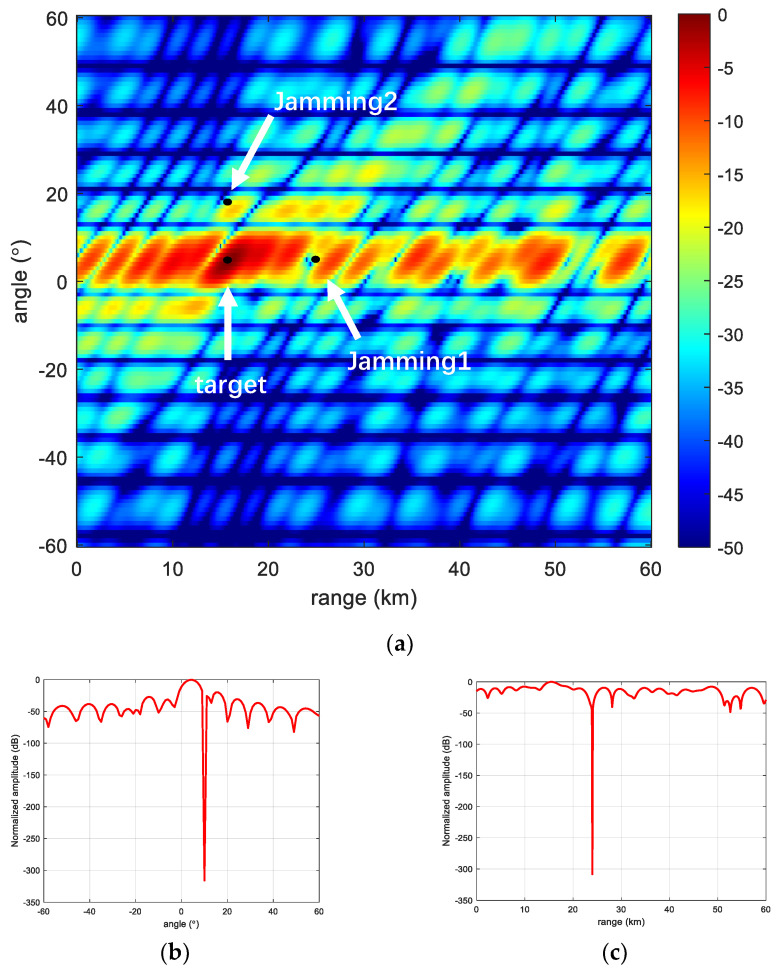
Log-FDA performance. (**a**) Log-FDA; (**b**) Range-dimensional slicing; and (**c**) Angle-dimensional slicing.

Figure 9 shows the beamforming performance of Log-FDA under MVDR conditions. It can be seen that the main flap of the Log-FDA beam becomes very wide and the position of the peak of the main flap is shifted, which indicates that Log-FDA does not adapt well to the environment where jamming is present.

**Figure 10:**
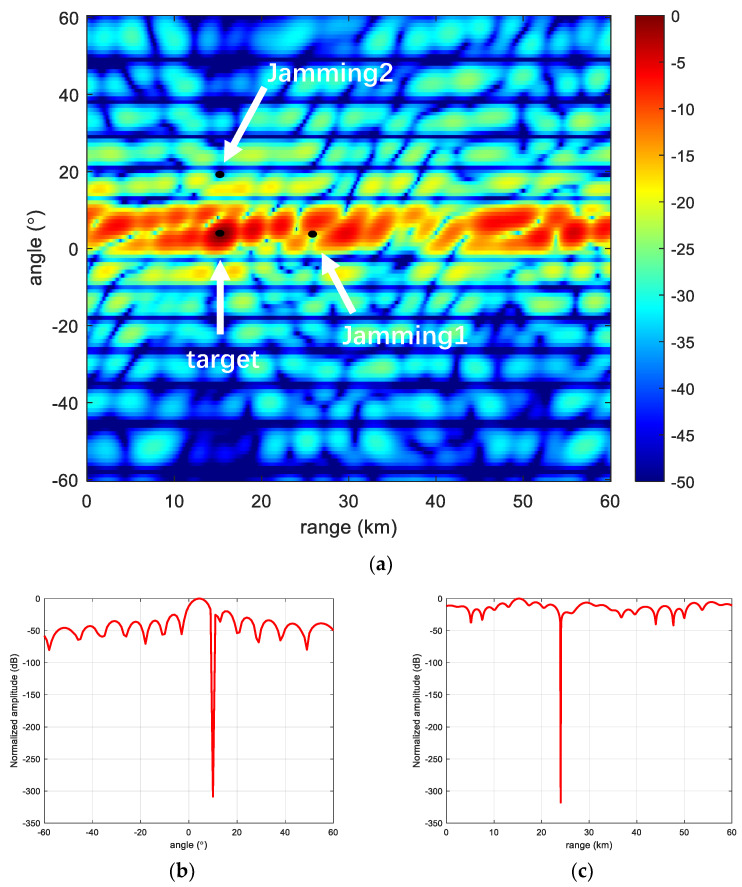
HL-FDA performance. (**a**) HL-FDA; (**b**) Range-dimensional slicing; and (**c**) Angle-dimensional slicing.

**Figure 11:**
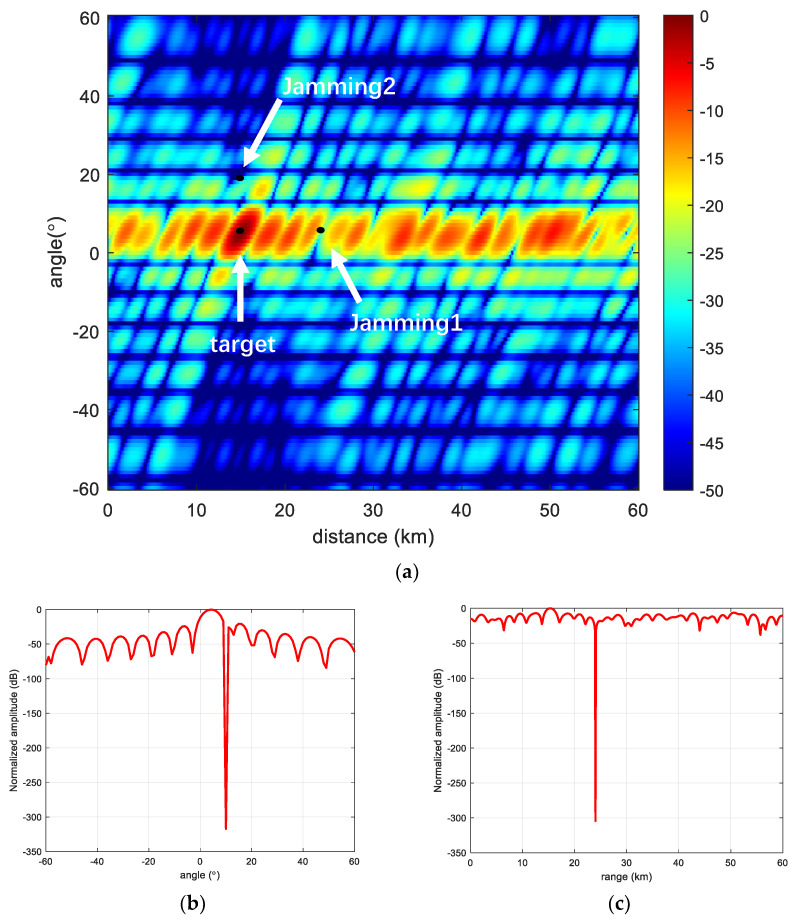
Proposed scheme performance. (**a**) The proposed scheme; (**b**) Range-dimensional slicing; and (**c**) Angle-dimensional slicing.

**Figure 12:**
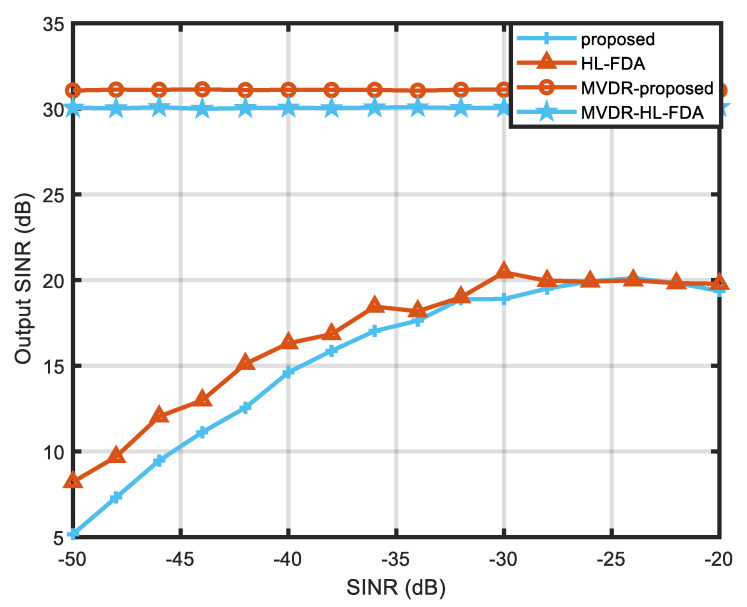
Comparison of output SINR.

Figure 10 and Figure 11 show the beamforming performance of HL-FDA under MVDR conditions. It can be seen that both HL-FDA and the proposed scheme can adapt well to the MVDR algorithm and can form zero traps at jamming locations in the presence of jamming in space. By observing the slice plots, it can be seen that the proposed scheme has a higher side flap in the range dimension but a narrower main flap width, which can better suppress jamming close to the main flap, which is one of the challenges faced by radar detection. Figure 12 shows the comparison of the output SINR in the presence and non-existence of MVDR conditions. It can be seen from the figure that the method in this paper has certain advantages over the traditional linear frequency bias method, which indicates that it is more applicable to the MVDR algorithm.

## 6. Experimental Results and Discussion

In this paper, the coupling problem of FDA radar is solved using the FDA-MIMO system, a new nonlinear frequency offset scheme is proposed, and its anti-interference performance is verified relative to several other frequency offset schemes using the MVDR algorithm. Considering that most of the current studies focus on the optimization using fixed window function weighting, this paper utilizes the Chebyshev window function to optimize the FDA frequency offset for the first time. Compared with the currently proposed HL-FDA algorithm and Ham-FDA algorithm, the algorithm proposed in this paper has better performance with the correct selection of parameters and better anti-interference performance when there is interference in space. However, since this method requires the selection of parameters, it may require more computation time in practical applications.

## Data Availability

All data used in this paper can be obtained by contacting the authors of this study.
